# Supplementary material for: The Effects of Mono- and Bivalent Linear Alkyl Interlayer Spacers on the Photobehavior of Mn(II)-Based Perovskites
Source: Int J Mol Sci. 2023 Feb 7;24(4):3280. doi: 10.3390/ijms24043280 (PMC9967457; doi:10.3390/ijms24043280)
Supplement: Supplementary file 1 [file ijms-24-03280-s001.zip › ijms-2191169-supplementary.pdf]

The Effects of Mono- and Bivalent Linear Alkyl Interlayer Spacers on the Photobehavior of Mn(II)-Based Perovskites

Soumyadipta Rakshit [1,†], Alicia Maldonado Medina [1,†], Luis Lezama [2], Boiko Cohen [1,*] and Abderrazzak Douhal [1,*]

[1] Departamento de Química Física, Facultad de Ciencias Ambientales y Bioquímica and INAMOL, Universidad de Castilla-La Mancha, 45071 Toledo, Spain
[2] Departamento de Química Orgánica e Inorgánica, Facultad de Ciencia y Tecnología, Universidad del País Vasco, UPV/EHU, Bº Sarriena s/n, 48940 Leioa, Spain
* Correspondence: boyko.koen@uclm.es (B.C.); abderrazzak.douhal@uclm.es (A.D.)
† These authors contributed equally to this work.

Supporting Information

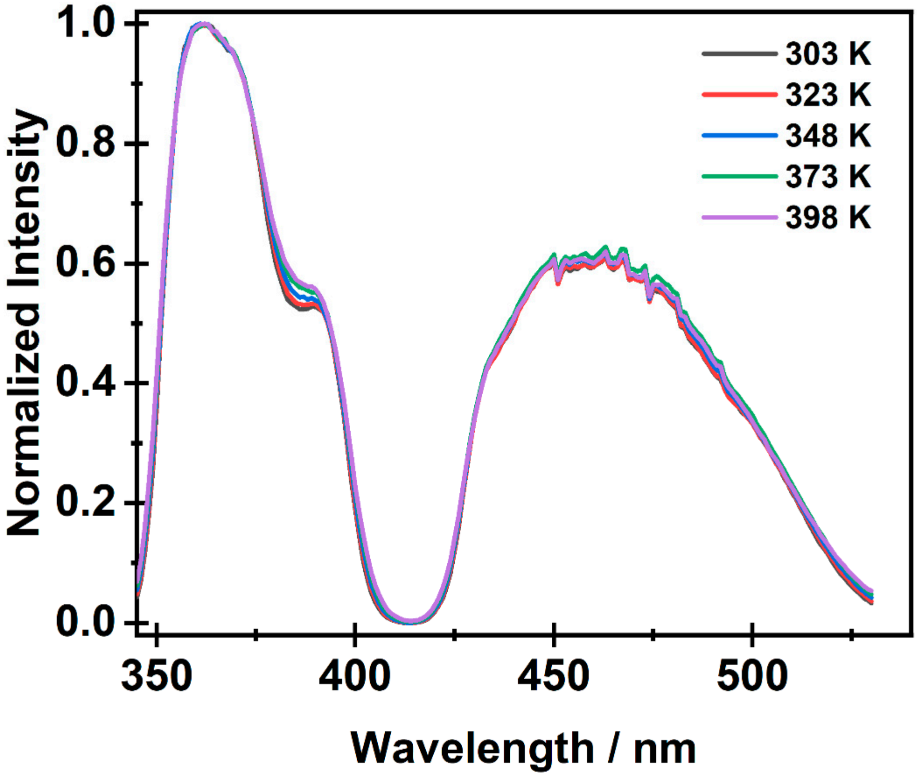

Figure S1. Normalized excitation spectra of P2 collected at the indicated temperatures. The emission wavelength is 550 nm.

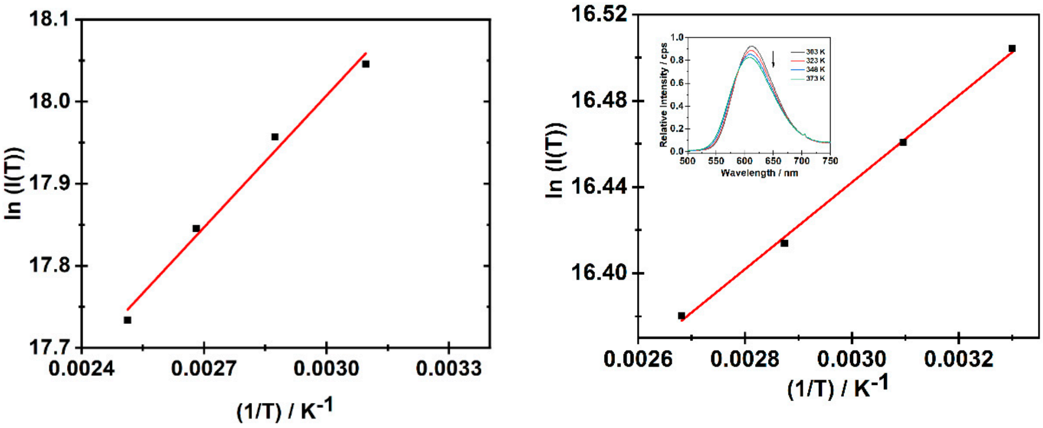

(**A**) (**B**)

Figure S2. Temperature dependence of the maximum emission intensity (**A**) at 550 nm for P2 from Figure 4 in the main text and (**B**) at 610 nm for P1 from the inset. Inset: Temperature dependence of the emission spectrum of P1 following excitation at 450 nm. The solid lines are the best fit using Equation 1 in the main text (R^2 = 0.98719 for P2 and R^2 = 0.9942).

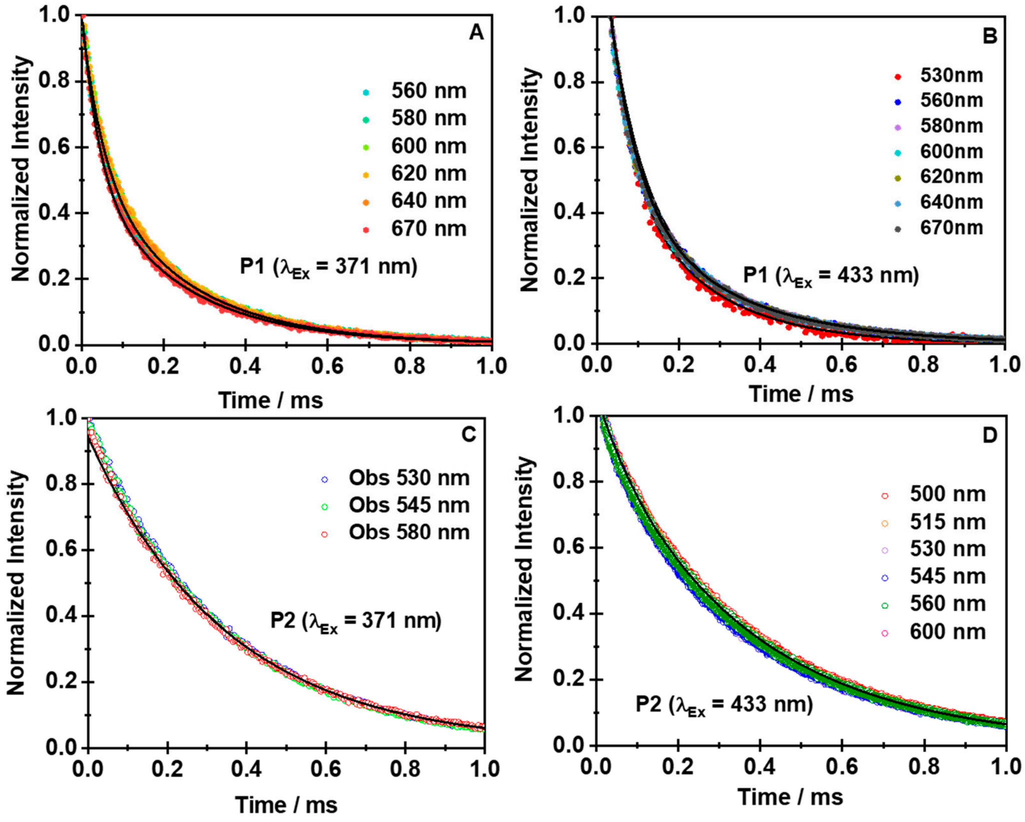

Figure S3. Comparison of the photoluminescence decays of P1/PMMA (**A**,**B**) and P2/PMMA (**C**,**D**) at the indicated wavelengths of observation following excitation at 371 nm (**A**,**C**) and 433 nm (**B**,**D**). Tables S1A–D give the related fitting parameters.

Table S1. (**A**) Values of the time constants (τ_i), normalized (to 100) pre-exponential factors (a_i) to fit the emission decays of P1/PMMA sample upon excitation at 371 nm and observation at the indicated emission wavelength (Figure S2A). (**B**) Values of the time constants (τ_i), normalized (to 100) pre-exponential factors (a_i) to fit the emission decays of P1/PMMA sample upon excitation at 433 nm and observation at the indicated emission wavelength (Figure S2B). (**C**) Values of the time constants (τ_i), normalized (to 100) pre-exponential factors (a_i) to fit the emission decays of P2/PMMA sample upon excitation at 371 nm and observation at the indicated emission wavelength (Figure S2C). (**D**) Values of the time constants (τ_i), normalized (to 100) pre-exponential factors (a_i) to fit the emission decays of P2/PMMA sample upon excitation at 433 nm and observation at the indicated emission wavelength (Figure S2D).

(**A**)

λ_{obs}	τ_1 (ms)	a_1	τ_2 (ms)	a_2
560 nm	0.070	51	0.27	49
580 nm	0.063	55	0.26	45
600 nm	0.074	50	0.25	50
620 nm	0.083	48	0.25	52
640 nm	0.084	53	0.27	47
670 nm	0.072	51	0.24	49

(**B**)

λ_{obs}	τ_1 (ms)	a_1	τ_2 (ms)	a_2
530 nm	0.078	55	0.22	45
560 nm	0.076	53	0.26	47
580 nm	0.081	54	0.25	46
600 nm	0.083	53	0.26	47
620 nm	0.080	53	0.27	47
640 nm	0.080	54	0.27	46
670 nm	0.070	51	0.24	49

(**C**)

λ_{obs}	τ_1 (ms)	a_1	τ_2 (ms)	a_2
530 nm	0.102	12	0.37	88
545 nm	0.102	11	0.37	89
580 nm	0.102	10	0.37	90

(**D**)

λ_{obs}	τ_1 (ms)	a_1	τ_2 (ms)	a_2
500 nm	0.1	10	0.37	90
515 nm	0.1	10	0.37	90
530 nm	0.1	11	0.37	89
545 nm	0.1	9	0.37	91
560 nm	0.1	10	0.37	90
580 nm	0.1	12	0.37	88
600 nm	0.1	12	0.37	88
